# Outcome of COVID-19 in Kidney Transplant Recipients Through the SARS-CoV-2 Variants Eras: Role of Anti-SARS-CoV-2 Monoclonal Antibodies

**DOI:** 10.3389/ti.2022.10721

**Published:** 2022-10-04

**Authors:** Matthaios Papadimitriou-Olivgeris, Ana Cipriano, Nicolas Guggisberg, Marie Kroemer, Jonathan Tschopp, Oriol Manuel, Dela Golshayan

**Affiliations:** ^1^ Infectious Diseases Service, Lausanne University Hospital, University of Lausanne, Lausanne, Switzerland; ^2^ Transplantation Center, Lausanne University Hospital, University of Lausanne, Lausanne, Switzerland; ^3^ Pharmacy Department, Lausanne University Hospital, University of Lausanne, Lausanne, Switzerland

**Keywords:** COVID-19, kidney transplantation, vaccination, outcome, monoclonal antibodies, SARS-CoV-2

## Abstract

Kidney transplant recipients (KTR) are at increased risk for COVID-19-associated complications. We aimed to describe the evolving epidemiology and outcome of PCR-documented SARS-CoV-2 infection in KTR followed at our institution from March 2020 to May 2022. The primary endpoint was hospitalization for COVID-19-related symptoms or death within 28 days from diagnosis. Overall, 243 cases were included of which 68 (28%) developed the primary outcome. A significant decrease in the incidence of the primary outcome was observed (*p* < 0.001, *r* −0.342) during the study period. Anti-Spike monoclonal antibodies (mAbs) were administered as early treatment (within 5–7 days of onset of symptoms) in 101 patients (14 with casirivimab/imdevimab and 87 with sotrovimab). Among 145 patients who had received at least one vaccination dose before infection, 109 patients were considered as adequately vaccinated. Multivariate analysis revealed that the Charlson Comorbidity Index (*P* 0.001; OR 1.28, CI 1.11–1.48) was associated with the primary outcome, while early administration of mAbs (*P* 0.032; OR 0.39, CI 0.16–0.92) was associated with a better outcome, but not infection during the period of the omicron variant predominance or adequate vaccination.

## Graphical Abstract

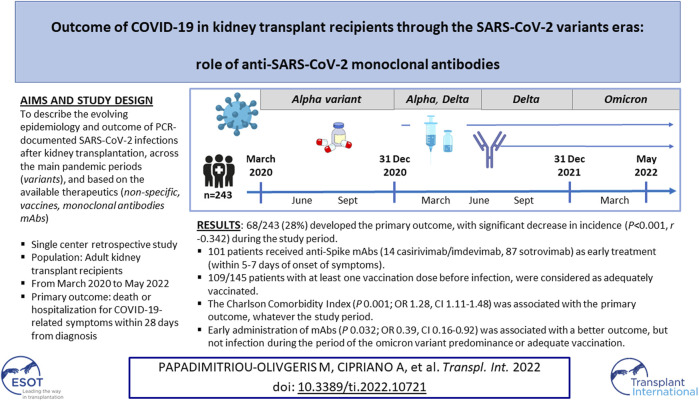

## Introduction

Kidney transplant recipients (KTR) represent a high-risk group for adverse outcomes of Coronavirus Disease 2019 (COVID-19) due to Severe Acute Respiratory Syndrome Coronavirus 2 (SARS-CoV-2), because of the burden of immunosuppression and the presence of comorbidities (obesity, diabetes mellitus, hypertension and cardiovascular diseases) (1, 2). In the first wave of the pandemic before specific anti-SARS-CoV-2 treatments were available, the overall mortality varied between centers, ranging from 19% to 50% (1–3). Acute kidney injury (AKI) was seen in 30%–89% of hospitalized patients and reported graft loss ranged between 4% and 11% (1, 2). These early studies usually included patients with moderate or severe disease, due to lack of testing for mild cases. As the pandemic evolved, subsequent studies showed an overall decrease of mortality, mostly attributed to earlier diagnosis (due to greater accessibility of testing), improvements in supportive care, and potential impact of preventive and therapeutic measures such as the use of corticosteroids, tocilizumab, anti-SARS-CoV-2 monoclonal antibodies (mAbs) and vaccination (4, 5).

Despite the availability of vaccination, solid organ transplant (SOT) recipients are known to elicit reduced humoral responses to mRNA SARS-CoV-2 vaccines, compared to the immunocompetent population (6–10). Variables described to be associated with lower or nonresponse to vaccination were older age, high dose corticosteroids, maintenance under triple immunosuppressive treatment and in particular the use of mycophenolic acid (MPA) (8). Some studies have additionally shown a higher risk for breakthrough COVID-19 in vaccinated SOT recipients as compared to the general population, although vaccinated patients had lower rates of hospitalization as compared to unvaccinated KTR (11, 12). The administration of early treatment with mAbs (casirivimab/imdevimab and sotrovimab) targeting the spike protein of SARS-CoV-2 has been used for high-risk patients with mild to moderate COVID-19, with promising results by reducing morbidity and mortality (13, 14). However, data on the efficacy of mAbs in the KTR population remain scarce, especially regarding sotrovimab (14–17). Some case-control studies performed in KTR showed that the administration of mAbs halted the progression of COVID-19 symptoms and decreased the number of hospitalizations related to COVID-19, with a good safety profile (15–18). In Switzerland, two mAbs became available in 2021: casirivimab/imdevimab and sotrovimab.

In this study, we aim to describe the evolving epidemiology of SARS-CoV-2 infections in Swiss KTR since the beginning of the pandemic, to assess the overall morbidity and mortality as well as the potential beneficial impact of anti-SARS-CoV-2 vaccination and mAbs on patients and grafts outcomes.

## Patients and Methods

### Study Design

This observational retrospective study was conducted at the Lausanne University Hospital (Lausanne, Switzerland), a 1500-bed tertiary care hospital and one of the six kidney transplantation centers in Switzerland. Our institution performs around 60 kidney transplantations per year and regularly follows around 1000 KTR. The study was approved by the institutional ethics review board (Swissethics Project-ID 2022-00324) for the retrospective use of clinical data.

### Patients

All adult (≥18 years old) KTR followed at our Transplantation Center who were diagnosed with a microbiologically-proven SARS-CoV-2 infection by real-time PCR between March 1st, 2020 and May 20th, 2022, were included in the analysis. Subsequent episodes of COVID-19 were included if they occurred at least 3 months after the previous one, based on reappearance of typical COVID-19 symptoms and *de novo* positive SARS-CoV-2 real-time PCR. Patients that had previously refused the institution’s general consent and those with graft loss (re-initiation of dialysis at the time of the study) were excluded. Patients were identified by the preexistent database including all KTR followed at our center. All patients were instructed to contact the transplantation center in case of COVID-19-compatible symptoms and following a positive antigenic test or PCR for SARS-CoV-2 irrespective of symptoms. Nephrologists responsible for the care of patients in other associated centers were additionally instructed to communicate with our center in the event of a positive case. Data were prospectively collected for all cases of COVID-19 in KTR in a secured database.

### Immunosuppressive Protocols

Depending on their immunological risk, KTR received basiliximab or anti-thymocyte globulins induction therapy (Thymoglobulin®). Maintenance immunosuppressive protocol generally consisted of the combination of a calcineurin inhibitor (CNI; mainly tacrolimus, TAC), mycophenolic acid (MPA), and prednisone following a tapering protocol during the first year. Beyond the first year, prednisone (5 mg/day) was only maintained in high immunological risk recipients. TAC doses were adjusted according to therapeutic drug monitoring and MPA according to digestive and haematological tolerability. All patients received co-trimoxazole prophylaxis during the first 6 months, and valgancyclovir or valacyclovir during the first 3 to 6 months according to donor/recipient serostatus.

### Management of Patients With COVID-19

Prevention and treatment of COVID-19 in KTR varied over time according to the availability of the different drugs and vaccines. From March 2020 to June 2020, only investigational drugs were used *via* the inclusion in clinical trials (hydroxychloroquine, lopinavir, remdesivir). Since June 2020, dexamethasone was used in all patients needing supplemental oxygen therapy. Tocilizumab was administered in selected patients not responding to dexamethasone. Remdesivir was not used in hospitalized patients on a routine basis. The vaccination campaign started in January 2021 and KTR were considered as a priority group for vaccination. Two doses of an mRNA vaccine (mRNA-1273 or BNT162b2) were proposed initially, with a third dose proposed from September 2021. Casirivimab/imdevimab (2400 mg) was available since July 2021. Sotrovimab (500 mg) was available in Switzerland since September 2021, although it was used at our institution only from end of December 2021, based on data regarding the reduced activity on the omicron variant of casirivimab/imdevimab as compared to sotrovimab (19, 20). Anti-Spike mAbs were proposed to all KTR with documented mild or moderate COVID-19 within 5–7 days of onset of symptoms (considered in this study as “early treatment”). From March 15th^,^ 2022, the dose of administered sotrovimab was doubled to increase its activity against the predominant omicron BA.2 variant (21). In addition, casirivimab/imdevimab was used in selected patients with severe COVID-19 and negative SARS-CoV-2 serology, according to the Recovery study (22). In this case, we used the term “late treatment” with mAbs. Following a positive SARS-CoV-2 test, MPA dosage was reduced by 50% or even stopped depending on the severity of the disease and/or concomitant administration of high dose corticosteroids. TAC trough levels were also decreased by around 30%.

### Outcomes and Data Collection

The primary outcome was death or hospitalization for COVID-19-related symptoms within 28 days from the diagnosis of infection. The secondary outcome was defined as need for oxygen therapy within 28 days. Data regarding demographics (age, sex), comorbidities, transplantation characteristics (date of transplantation, immunosuppression, graft function), vaccination status (BNT162b2 or mRNA-1273), SARS-CoV-2 serology, specific anti-SARS-CoV-2 treatments including mAbs (casirivimab/imdevimab and sotrovimab), and complications were collected in the patients’ electronic health records. SARS-CoV-2 serology (IgG) was performed using a previously described Luminex-based (Luminex Corp) assay quantifying antibody binding to the trimeric form of the SARS-CoV-2 S-protein and divided by the negative control; a ratio of ≥5.9 was considered positive (23).

All data were collected, stored and managed using REDCap electronic data capture tools hosted at Lausanne University Hospital. REDCap (Research Electronic Data Capture) is a secure, web-based software platform designed to support data capture for research studies (24, 25).

### Definitions

The date of the first positive SARS-CoV-2 PCR was defined as infection onset. Acute kidney injury (AKI) was defined according to the 2012 Kidney Disease Improving Global Outcome (KDIGO) guidelines. Reduction of immunosuppressive treatment was defined as at least 50% MPA or 30% TAC dose decrease. Adequate vaccination was defined as having received three doses before infection or developing infection within 4 months after two doses. By using the data from the Swiss Federal Office of Public Health (26) that monitored the circulation and prevalence of SARS-CoV-2 variants, we divided the study in four periods: Period 1 (March to December 2020): pre-vaccination period, with the initial virus or alpha variant; Period 2 (January to June 2021): vaccination available but before mAbs, with the alpha and delta variants; Period 3 (July to December 2021): vaccination available and mAbs, with the delta variant; and Period 4 (January to May 2022): vaccination available and mAbs, with the omicron variant.

### Statistical Analyses

The SPSS version 26.0 (SPSS, Chicago, IL, United States) software was used for data analysis. Categorical variables were analyzed using the *chi*-square or Fisher exact test and continuous variables with Mann-Whitney *U* test. Two multivariate logistic regression analyses were performed with primary and secondary outcomes, respectively, as the dependent variables. Four variables from the univariate analysis with *p* < 0.05 (Charlson Comorbidity Index, adequate vaccination, mAbs as early treatment, Period 4) that did not contribute to multicollinearity were used in multivariate logistic regression model. Odds ratios (OR) and 95% confidence intervals (CI) were calculated to evaluate the strength of any association. The primary and secondary outcomes trends during the pandemic periods were assessed using Spearman’s correlation analysis. All statistic tests were 2-tailed and *p* < 0.05 was considered statistically significant.

## Results

### Patients Characteristics

Overall, 246 KTR with at least one episode of COVID-19 were identified, for whom 243 episodes were included in the study corresponding to 237 patients (6 patients had two episodes of COVID-19 during the study period). Among the 9 patients that were excluded, 4 patients were excluded for refusal of general consent and 5 due to graft loss at the time of study initiation. Patients’ characteristics according to the time-period of SARS-CoV-2 diagnosis are shown in Table 1. Overall, there was no significant difference in the demographic characteristics of the infected patients during the different pandemic periods. The majority of patients were middle-aged men with 22% suffering from obesity, 24% from diabetes, and/or 14% from coronary heart disease, representative of the general KTR population. The majority were on CNI-based (mainly TAC) triple immunosuppressive therapy, including prednisone (70%) and MPA (77%). No patient was on belatacept maintenance immunosuppressive therapy and only a minority of the study population (4%) had received T- or B-cell depleting agents in the previous year before suffering from COVID-19, and one patient received eculizumab every 3 weeks for the treatment of recurrent glomerulonephritis.

**TABLE 1:** Patients’ characteristics depending on the period of SARS-CoV-2 diagnosis.

Characteristics	Period 1 (*n* = 63)	Period 2 (*n* = 24)	Period 3 (*n* = 41)	Period 4 (*n* = 115)	All episodes (*n* = 243)
Demographics					
Male sex	40 (64%)	18 (75%)	27 (66%)	72 (63%)	157 (65%)
Age (years)	62 (50–70)	60 (48–68)	55 (43–67)	57 (43–66)	58 (45–68)
Co-morbidities					
Coronary heart disease	8 (13%)	3 (13%)	4 (10%)	19 (17%)	34 (14%)
Congestive heart failure	1 (2%)	1 (4%)	2 (5%)	5 (4%)	9 (4%)
Chronic obstructive pulmonary disease	2 (3%)	2 (13%)	2 (5%)	6 (5%)	13 (5%)
Diabetes mellitus	16 (25%)	7 (29%)	10 (24%)	25 (22%)	58 (24%)
Malignancy (solid organ or hematologic)	9 (14%)	1 (4%)	0 (0%)	5 (4%)	15 (6%)
Obesity	10 (16%)	7 (29%)	10 (24%)	26 (23%)	53 (22%)
Charlson Comorbidity Index	4 (3–6)	5 (3–6)	4 (2–5)	4 (2–6)	4 (2–6)
Transplantation data					
Years from transplantation	6 (3–12)	7 (3–12)	6 (3–11)	6 (3–11)	6 (3–12)
Combined kidney and other organ transplantation	3 (5%)	1 (4%)	3 (7%)	7 (6%)	14 (6%)
Immunosuppressive treatment					
Tacrolimus	50 (79%)	23 (96%)	39 (95%)	101 (88%)	213 (88%)
Cyclosporine	5 (8%)	0 (0%)	1 (2%)	6 (5%)	12 (5%)
Mycophenolic acid	42 (71%)	20 (83%)	32 (78%)	91 (79%)	188 (77%)
Azathioprine	2 (3%)	1 (4%)	7 (17%)	9 (8%)	19 (8%)
Prednisone	43 (68%)	16 (68%)	26 (63%)	84 (73%)	169 (70%)
Other	2 (3%)	1 (4%)	1 (2%)	8 (7%)	12 (5%)
Triple immunosuppressive treatment	31 (49%)	13 (54%)	25 (61%)	75 (65%)	144 (59%)
Rituximab or Thymoglobulin (within the last year)	2 (3%)	1 (4%)	1 (2%)	6 (5%)	10 (4%)
Vaccination status					
No vaccination	63 (100%)	18 (75%)	6 (15%)	11 (10%)	98 (40%)
One dose	0 (0%)	1 (4%)	2 (5%)	1 (1%)	6 (3%)
Two doses	0 (0%)	3 (13%)	23 (56%)	18 (16%)	44 (18%)
Three doses	0 (0%)	0 (0%)	10 (24%)	85 (74%)	95 (39%)
Adequate vaccination	0 (0%)	3 (13%)	15 (37%)	91 (79%)	109 (45%)
Serology before infection (among 103 episodes)^a^	—	—	6.5 (0.9–29.1)	28.9 (8.8–83.4)	27.0 (3.5–72.9)
Positive serology	—	—	11 (61%)	66 (80%)	83 (77%)
SARS-CoV-2 infection					
Community	59 (94%)	20 (83%)	40 (98%)	111 (97%)	230 (95%)
Nosocomial	4 (6%)	4 (17%)	1 (2%)	4 (4%)	13 (5%)
Reduction of immunosuppression	23 (37%)	6 (25%)	16 (39%)	8 (7%)	53 (22%)
Monoclonal antibodies (as early treatment)	0 (0%)	0 (0%)	19 (46%)	82 (71%)	101 (42%)
Casirivimab/imdevimab	0 (0%)	0 (0%)	14 (34%)	0 (0%)	14 (6%)
Sotrovimab	0 (0%)	0 (0%)	5 (12%)	82 (71%)	87 (36%)
Hospitalization (within 28 days)	34 (54%)	8 (33%)	15 (37%)	20 (17%)	77 (32%)
Hospitalization due to COVID-19	31 (52%)	6 (30%)	14 (34%)	15 (15%)	66 (30%)
Need for oxygen therapy (secondary outcome)	21 (33%)	5 (21%)	12 (29%)	6 (5%)	44 (18%)
Non-mechanical ventilation or Optiflow	7 (11%)	4 (17%)	4 (10%)	2 (2%)	17 (7%)
Intensive Care Unit hospitalization	8 (13%)	4 (17%)	6 (15%)	2 (2%)	20 (8%)
Mechanical ventilation	4 (6%)	2 (8%)	4 (10%)	1 (1%)	11 (5%)
Treatment					
Convalescent plasma	2 (3%)	4 (17%)	0 (0%)	2 (2%)	8 (3%)
Lopinavir/ritonavir	2 (3%)	0 (0%)	0 (0%)	0 (0%)	2 (1%)
Hydroxychloroquine	3 (5%)	0 (0%)	0 (0%)	0 (0%)	3 (1%)
Remdesivir	3 (5%)	0 (0%)	0 (0%)	0 (0%)	3 (1%)
Tocilizumab	0 (0%)	2 (8%)	3 (7%)	0 (0%)	5 (2%)
Casirivimab/imdevimab (as late treatment)	0 (0%)	0 (0%)	6 (15%)	0 (0%)	6 (3%)
Dexamethasone	16 (25%)	5 (21%)	11 (27%)	6 (5%)	38 (16%)
Death (within 28 days)	5 (8%)	1 (4%)	1 (2%)	1 (1%)	8 (3%)
Primary outcome (death or hospitalization for infection-related symptoms or complications)	32 (51%)	7 (29%)	14 (34%)	15 (13%)	68 (28%)
Acute complications					
Acute kidney injury	7 (11%)	4 (17%)	5 (12%)	3 (3%)	19 (8%)
Community-acquired pneumonia	4 (6%)	3 (13%)	4 (10%)	3 (3%)	14 (6%)
Renal function at 28 days					
Creatinine increase >15% from baseline (among 175 episodes)	6 (13%)	5 (28%)	5 (15%)	8 (10%)	24 (14%)
Creatinine increase ≥ AKIN stage I (among 175 episodes)	5 (11%)	4 (22%)	5 (15%)	3 (4%)	17 (10%)
*De novo* donor‐specific anti-HLA antibodies (among 112 episodes)	3 (7%)	0 (0%)	1 (4%)	1 (2%)	5 (4%)

Data are depicted as number and percentage or median and Q1-3.

a Six cases that belong in Periods 1 and 2 are not included.

### Outcomes

In total, 77 patients (32%) were hospitalized within 28 days from diagnosis; 66 patients were hospitalized due to COVID-19 symptoms and 44 patients needed oxygen therapy. Eight patients (3%) died within 28 days from the diagnosis of infection. Sixty-eight patients (28%) developed the primary outcome (hospitalization for COVID-19-related symptoms or death within 28 days from infection diagnosis) and 44 (18%) the secondary endpoint (need for oxygen therapy within 28 days). Hospitalization for COVID-19-related symptoms or death was seen in 45% (39/87) of patients during Period 1 and 2 and 19% (29/156) of patients during Period 3 and 4. A significant decrease in the incidence of primary (*p* < 0.001, *r* −0.342) and secondary outcomes (*p* < 0.001, *r* −0.311) was observed during the consecutive study periods. Overall, AKI (≥ AKIN stage I) was observed in 8% of KTR, and the same proportion (10–14%) of patients had persisting moderate to severe graft dysfunction at 28 days. Four patients lost their graft and returned to dialysis following severe COVID-19. Among 112 patients in whom anti-HLA Abs could be screened after the episode of SARS-CoV-2 infection, 5 (4%) developed *de novo* donor-specific anti-HLA Abs (DSA). There was however no episode of acute cellular or antibody-mediated rejection that could be associated with the infection.

### Use of mAbs

In total, mAbs were administered as early treatment in 101 patients (14 with casirivimab/imdevimab and 87 with sotrovimab), and 6 (3%) additional patients received casirivimab/imdevimab as a late treatment (Table 1). Double dose of sotrovimab was administered in 17 patients, of whom two were hospitalized due to COVID-19 symptoms and one needed oxygen therapy.

### Vaccination and SARS-CoV-2 Serostatus

**FIGURE 1:**
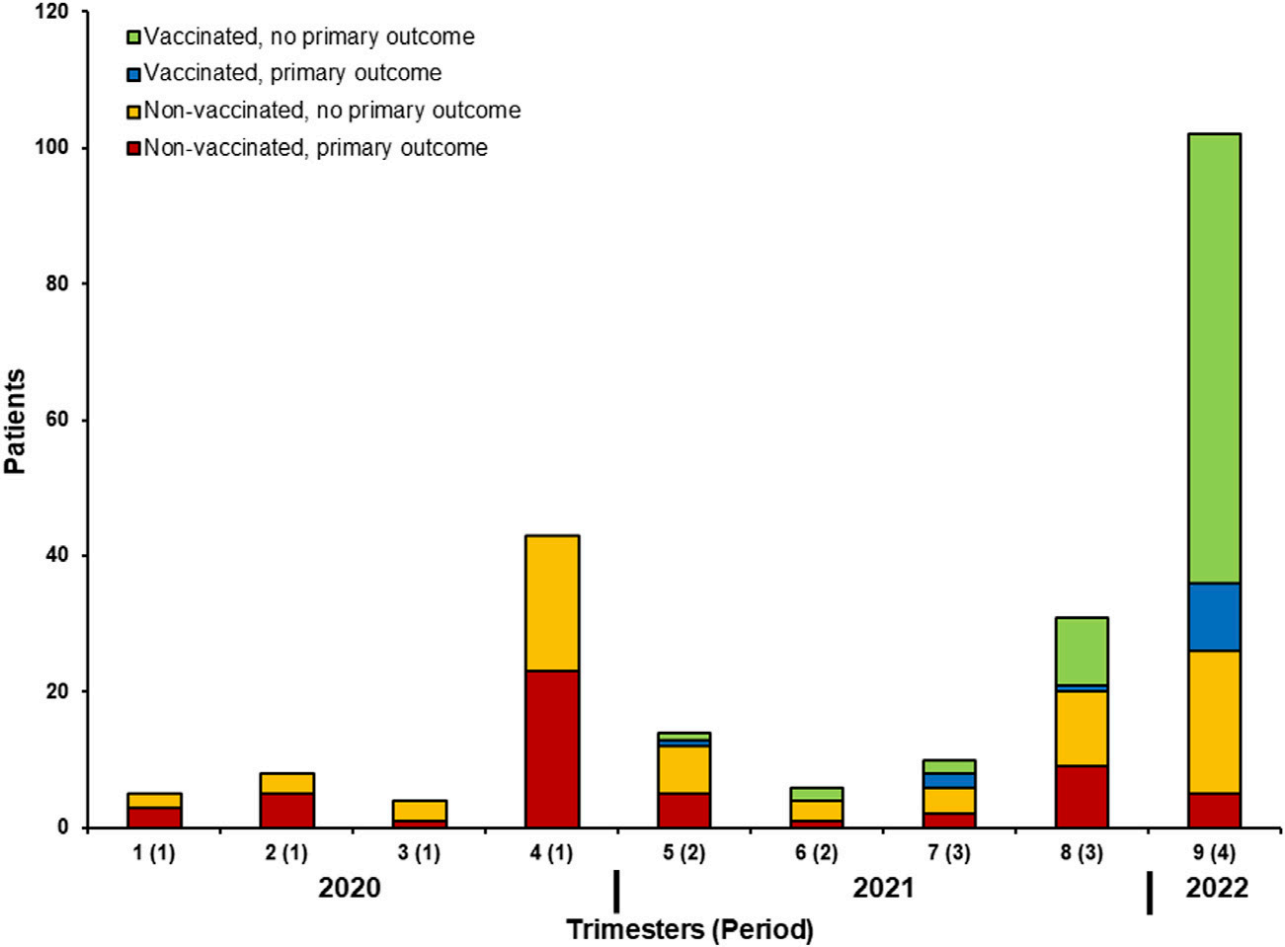
Number of patients with the primary outcome depending on adequate vaccination and timing of SARS-CoV-2 infection.

**FIGURE 2:**
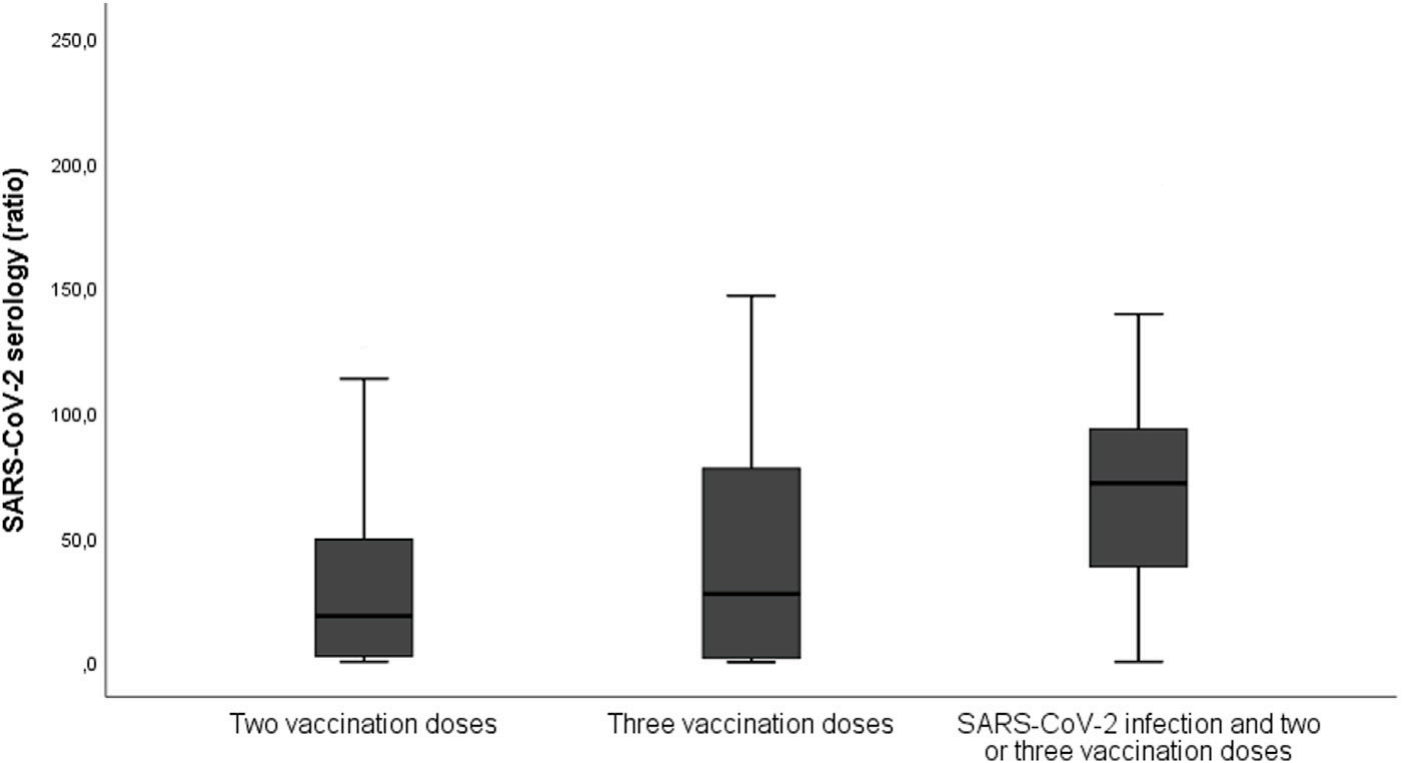
Results of SARS-CoV-2 serology depending on its timing (after vaccination and/or SARS-CoV-2 infection). The serology was performed using Luminex-based assay quantifying antibody (IgG) binding to the trimeric form of the SARS-CoV-2 S-protein and divided by the negative control; a ratio of ≥5.9 was considered positive. The median ratio for patients with two vaccination doses without prior infection was 18.5, those with three vaccination doses without prior infection was 27.4, and for those with two or three doses and prior SARS-CoV-2 infection the ratio was 71.7.

Among 145 patients that had received at least one vaccination dose before infection, 109 (45% of all infection episodes) were considered as adequately vaccinated. Figure 1 shows the number of patients with the primary outcome depending on adequate vaccination and timing of SARS-CoV-2 infection. Serology was performed in 109 patients at the time of SARS-CoV-2 infection diagnosis and it was positive in 83 (76%). Among 108 patients for whom serology was performed after two or three doses (without documented prior infection), 83 (77%) had positive serology. Figure 2 shows the results of SARS-CoV-2 serology depending on the timing of sampling (after vaccination and/or SARS-CoV-2 infection). Serology results of patients with two or three vaccine doses and with prior SARS-CoV-2 infection (median ratio of 71.7) were significantly higher (*p* < 0.001) than for those who received two (median ratio of 18.5) or three doses (median ratio of 27.4) without prior infection.

### Variables Associated With the Primary and Secondary Outcomes

Multivariate analysis revealed that the Charlson Comorbidity Index (*P* 0.001; OR 1.28, CI 1.11–1.48) was associated with the primary outcome, while administration of mAbs as early treatment (*P* 0.032; OR 0.39, CI 0.16–0.92) was associated with a better outcome (Table 2). Of note, adequate vaccination and infection during Period 4 were associated with improved primary outcome in the univariate analysis, but this was not confirmed in the multivariate analysis. In the multivariate analysis for the secondary outcome (hospitalization for need of oxygen), the Charlson Comorbidity Index (*P* 0.001; OR 1.30, CI 1.11–1.51) increased the risk of secondary outcome, while administration of mAbs as early treatment (*P* 0.009; OR 0.19, CI 0.06–0.66) was associated with a reduced risk for the secondary outcome. Similarly, adequate vaccination and infection during Period 4 were not associated with the secondary outcome.

**TABLE 2:** Univariate and multivariate analyses among patients with and without the primary outcome.

Characteristics	Univariate analysis			Multivariate analysis	
	No primary outcome (*n* = 175)	Primary outcome (*n* = 68)	*P*	OR (95% CI)	*P*
Demographics					
Male sex	115 (66%)	42 (62%)	0.563		
Age (years)	55 (42–67)	63 (52–69)	0.001		
Co-morbidities					
Coronary heart disease	23 (13%)	11 (16%)	0.541		
Congestive heart failure	5 (3%)	4 (6%)	0.271		
Chronic obstructive pulmonary disease	6 (3%)	7 (10%)	0.033		
Diabetes mellitus	36 (21%)	22 (32%)	0.053		
Malignancy (solid organ or hematologic)	7 (4%)	8 (12%)	0.024		
Obesity	37 (21%)	16 (24%)	0.686		
Charlson Comorbidity Index	4 (2–5)	5 (4–7)	<0.001	1.28 (1.11–1.48)	0.001
Transplantation data					
Years from transplantation	7 (3–12)	5 (2–12)	0.492		
Combined kidney and other organ transplantation	9 (5%)	5 (7%)	0.507		
Immunosuppressive treatment					
Tacrolimus	154 (88%)	59 (87%)	0.793		
Cyclosporine	9 (5%)	3 (4%)	1.000		
Mycophenolic acid	137 (78%)	51 (75%)	0.610		
Azathioprine	16 (9%)	3 (4%)	0.291		
Prednisone	121 (69%)	48 (71%)	0.826		
Other	9 (5%)	3 (4%)	1.000		
Triple immunosuppressive treatment	110 (63%)	34 (50%)	0.067	0.83 (0.44–1.48)	0.574
Rituximab or Thymoglobulin (within the last year)	8 (5%)	2 (3%)	0.730		
Periods					
Period 1	31 (18%)	32 (47%)			
Period 2	17 (10%)	7 (10%)			
Period 3	27 (15%)	14 (21%)			
Period 4	100 (57%)	15 (22%)	<0.001^a^	0.60 (0.23–1.54)	0.288^a^
Vaccination status					
No vaccination	55 (31%)	43 (63%)			
One dose	4 (2%)	2 (3%)			
Two doses	31 (18%)	13 (19%)			
Three doses	85 (48%)	10 (15%)	<0.001^b^		
Adequate vaccination	95 (54%)	14 (21%)	<0.001	0.44 (0.18–1.09)	0.077
Serology before infection (among 109 episodes)	28.7 (6.4–81.1)	3.8 (0.6–36.2)	0.008		
Positive serology	77 (81%)	6 (46%)	0.005		
Monoclonal antibodies (as early treatment)	89 (51%)	12 (18%)	<0.001	0.39 (0.16–0.92)	0.032
Casirivimab/imdevimab	12 (7%)	2 (3%)	0.240		
Sotrovimab	77 (44%)	10 (15%)	<0.001		
Sotrovimab (double dose)	15 (9%)	2 (3%)	0.164		

Data are depicted as number and percentage or median and Q1-3.

a Comparison of Period 4 to all other periods.

b Comparison between patients having received three doses and those that have not.

## Discussion

The first aim of this study was to describe the epidemiology of SARS-CoV-2 infection in at-risk immunosuppressed KTR, based on the evolution of the pandemic and the availability of preventive and therapeutic measures. Interestingly, we observed that adverse outcomes related to COVID-19 (death, SARS-CoV-2-related hospitalizations) declined over time (51% in Period 1 to 13% in Period 4), similar to what has been described in the general population (27). As the patients’ demographic characteristics did not significantly differ over time, these outcomes could be mainly explained by the pathogenicity of the prevalent variants during the different periods of the study, together with better preventive and therapeutic management of KTR with COVID-19. An important finding of this study is that administration of mAbs as ealry treatment was associated with lower rates of adverse outcomes (mortality or hospitalization). Only 12% of patients who received mAbs were hospitalized for SARS-CoV-2-related symptoms or died within 28 days of the diagnosis of infection. These results are similar to what was previously reported in two studies using bamlanivimab or casirivimab/imdevimab in SOT recipients (16, 28), although another study did not confirm this positive impact in immunosuppressed SOT recipients (29). To the best of our knowledge, this is the largest study in kidney transplantation that describes patients’ management and outcomes over time during the 2 years of SARS-CoV-2 pandemic. In addition, we report a beneficial effect of sotrovimab administration in KTR, with a significant reduction of deaths or hospitalizations within 28 days of infection diagnosis. Our results corroborate a recent publication that describes the benefit of an early use of mAbs in KTR with a mild form of COVID-19 (30). This is also the first study, reporting the preventive use of a double dose of sotrovimab against omicron BA.2 variant, with only one patient (6%) subsequently admitted for oxygen therapy. While in Switzerland mAbs are used only as an early treatment, neutralizing anti–SARS-CoV-2 mAbs such as casirivimab/imdevimab were used as pre-exposure prophylaxis in SOT recipients with weak or no humoral response after vaccination (3 doses of an mRNA vaccine). This latter strategy was shown to be efficient in preventing COVID-19 incidence in SOT, compared to untreated controls (17).

An important observation in our study is that the humoral response to adequate vaccination was higher than previously reported (14%–38%) among KTR (8, 31, 32). A possible explanation could be the different testing methods used and the absence of a well-established protective antibody titer. For the chosen cut-off of positivity defined at a ratio >5.90, the assay used in the present study has shown a sensitivity and specificity of 97% and 98%, respectively, in hospitalized patients (23). As compared to a healthy control population, the predictors of failure for SOT recipients to mount a humoral response were described to be higher age, need for high-dose corticosteroids during the last year, maintenance under triple immunosuppressive therapy, and a regimen that included MPA (8, 31). In our study, no factor among the studied ones was found to be associated with the humoral response in KTR.

Patients with an increased Charlson Comorbidity Index, incorporating age and comorbidities, had a higher risk of death or hospitalization within 28 days from infection diagnosis, whatever the study period. While in previous reports SOT recipients’ characteristics differed between the various waves of the SARS-CoV-2 pandemic, with higher rates of high-risk comorbidities (cardiovascular, pulmonary) in the earlier periods (4), no such difference was found in the present study. Thus, comorbidities did not play a role in the lower mortality observed in the later periods of our study.

The study has several limitations. First, it is a retrospective monocentric study including a relatively moderate number of patients. Second, there is a selection bias towards symptomatic patients, as paucisymptomatic or asymptomatic KTR that did not seek medical attention and did not have a PCR-documented infection were not included in the study. This bias should be minimal, since KTR were strongly advised to be tested and to contact their physician at the occurrence of the first symptoms. Third and more importantly, the study included patients during a 2-year period with a changing viral epidemiology, SARS-CoV-2 variants associated with diverse pathogenicity (33), and different therapeutic (mAbs) or preventive modalities (vaccination); all factors influencing the outcomes. We cannot exclude that some confounders were not adjusted in the multivariate analyses. Finally, viral sequencing was not routinely available, so that we used the period of infection as a proxy for the different variants, as done in other epidemiological studies (27). Thus, some misclassification cannot be excluded.

In conclusion, we observed a decrease in unfavorable outcomes of infected KTR in the last wave of the pandemic. Although these changes are probably due to a combination of factors, we identified the use of mAbs as the only measure significantly associated with a better outcome. Prospective studies are needed to better delineate the role of mAbs and vaccination in preventing COVID-19-associated complications in immunocompromised patients, particularly in the era of the new variants.

## Data Availability

The original contributions presented in the study are included in the article/supplementary material, further inquiries can be directed to the corresponding author.
